# Statistical reproducibility for pairwise *t*-tests in pharmaceutical research

**DOI:** 10.1177/09622802211041765

**Published:** 2021-12-02

**Authors:** Andrea Simkus, Frank PA Coolen, Tahani Coolen-Maturi, Natasha A Karp, Claus Bendtsen

**Affiliations:** 1 Department of Mathematical Sciences, 3057 Durham University, UK; 2 Data Sciences & Quantitative Biology, Discovery Sciences, 468087 R&D, AstraZeneca, Cambridge, UK

**Keywords:** Nonparametric predictive inference, pharmaceutical product development, statistical reproducibility, *t*-test,, Wilcoxon Mann–Whitney test

## Abstract

This paper investigates statistical reproducibility of the t -test. We formulate reproducibility as a predictive inference problem and apply the nonparametric predictive inference method. Within our research framework, statistical reproducibility provides inference on the probability that the same test outcome would be reached, if the test were repeated under identical conditions. We present an nonparametric predictive inference algorithm to calculate the reproducibility of the t -test and then use simulations to explore the reproducibility both under the null and alternative hypotheses. We then apply nonparametric predictive inference reproducibility to a real-life scenario of a preclinical experiment, which involves multiple pairwise comparisons of test groups, where different groups are given a different concentration of a drug. The aim of the experiment is to decide the concentration of the drug which is most effective. In both simulations and the application scenario, we study the relationship between reproducibility and two test statistics, the Cohen’s d and the p -value. We also compare the reproducibility of the t -test with the reproducibility of the Wilcoxon Mann–Whitney test. Finally, we examine reproducibility for the final decision of choosing a particular dose in the multiple pairwise comparisons scenario. This paper presents advances on the topic of test reproducibility with relevance for tests used in pharmaceutical research.

## Introduction

Reproducibility of tests is a complex issue, which is of importance in pharmaceutical research and development. Lack of reproducibility contributes to the high failure rate in the drug discovery process, increasing costs and decreasing efficiency.^1–5^ There are many factors which may lead to poor reproducibility, these include wrong or unsuitable statistical analysis of results or inadequate sample sizes,^4^ and also poor documentation and inappropriate models.^5^ This paper focuses only on the variability of statistical methods, which exists due to variability of data, not on further aspects of reproducibility. By its nature, it is attractive to consider reproducibility as a predictive inference problem.^6,7^ Predictive inference is about predicting future observations based on existing data. Assume that a test has been performed, and a test outcome, whether or not to reject the null hypothesis, has been reached. We define statistical reproducibility as the probability for the event that, if the test were repeated under identical circumstances and with the same sample size, the same test outcome would be reached.

Research on statistical reproducibility has been gaining importance for the past three decades. The first insights related to the topic of this paper were provided by Goodman,^8^ who highlighted a misconception regarding the p -value. He questioned the claim that a small p -value increases the credibility of the test result and argued that the replication probability may be smaller than expected. Although Goodman used the term replication probability rather than reproducibility probability, his definition is very similar to the definition of reproducibility adopted in this paper. He defined it as the probability of observing another statistically significant result in the same direction as the first one, if an experiment was repeated under identical conditions and with the same sample size. Senn^9^ agreed with Goodman that the p -value and replication probability are different measures and that inconsistency between test results from individual studies may be expected. However, he disagreed with Goodman’s claim that the p -value overstates the evidence against the null hypothesis.^8^ Senn pointed out that we should recognise a link between the p -values and replication probability. In this paper, we build further upon Goodman’s and Senn’s discussion and we provide more insights into statistical reproducibility.

In the literature, several other approaches to reproducibility probability have been presented. For example, De Capitani and De Martini^10–12^ consider the estimated power approach.^13^ They equate the reproducibility probability to the true power of a statistical test and they argue that ‘its estimation provides useful information to evaluate the stability of statistical test results’.^12^ They adopt Goodman’s definition of reproducibility probability, that is, the probability of obtaining the same test result in a second, identical experiment, but they consider it as an estimation problem instead of a prediction problem. Furthermore, they only consider reproducibility in case the null hypothesis is rejected in the test, while we provide predictive inference for reproducibility both if the null hypothesis is rejected or not rejected.

In this paper, we use the nonparametric predictive inference (NPI) framework for inference on reproducibility. NPI is a frequentist statistical approach, based on only few assumptions, and focused on future observations, which makes it a suitable methodology for inference on reproducibility. NPI has been applied in many areas, for example, in finance,^14^ system reliability,^15^ operations research^16^ and receiver operating characteristic analysis.^17^

The first application of NPI to test reproducibility was presented by BinHimd and Coolen,^18,6^ who explored NPI reproducibility for simple nonparametric tests, such as the Wilcoxon Mann–Whitney test (WMT), and they also developed NPI bootstrap, which is a computational implementation of NPI that is also employed in this paper. Alqifari and Coolen^19,20^ developed NPI reproducibility for tests on population quantiles and for a precedence test. Marques et al.^21^ studied reproducibility for likelihood ratio tests. NPI reproducibility has not yet been presented for the t -test, which is a common test used in pharmaceutical research. Moreover, to date NPI exploration has been mainly theoretical. This paper contributes to the literature by presenting NPI reproducibility for the t -test and its application in a real-world scenario.

The paper begins with a brief review of NPI and NPI bootstrap in section ‘Nonparametric predictive inference and bootstrap’. Section ‘NPI reproducibility for pairwise t -test’ presents an algorithm for calculating the reproducibility of the t -test for comparison of two groups (Algorithm 1), and we present the results of simulation studies to investigate the reproducibility of the t -test. Following the simulation study, a pre-existing pharmaceutical test scenario is introduced and the reproducibility of pairwise comparisons tests for this scenario is studied in section ‘NPI reproducibility for t -test applied to a pharmaceutical test scenario’. This test scenario investigates the optimal dose of a drug. Different doses of the treatment are given to members of different groups and pairwise comparisons are carried out on a recorded variable between adjacent doses. In sections ‘NPI reproducibility for pairwise t -test’ and ‘NPI reproducibility for t -test applied to a pharmaceutical test scenario’ we explore the relationship between two test statistics, namely Cohen’s d and the p -value, and NPI reproducibility. We explore the assumption that, if the original test statistic is close to the threshold value between rejection of the null hypothesis and non-rejection, then the test can be expected to be less reproducible than when the test statistic is further away from the threshold. We also briefly compare reproducibility of the t -test and the WMT.

Finally, a novel algorithm for calculating the reproducibility of the final decision based on multiple pairwise t -tests (Algorithm 2) is described and applied to the pharmaceutical test scenario in section ‘Reproducibility of the final decision based on multiple pairwise comparisons’. This final decision is of interest as in practice decisions are often based on more than one single statistical test; hence studying its reproducibility is important and to date has received little attention in the literature. The paper concludes with a summary of the findings and with formulation of future research topics in section ‘Concluding remarks’. All calculations have been done using R version 3.2.4, the code is available from the link https://tahanimaturi.com/rcodes/Rcodes-SMMR-May-2021.zip.

## Nonparametric predictive inference and bootstrap

NPI is based on Hill’s assumption $A_{\left(n\right)}$ , which is a post-data assumption that gives conditional probabilities for a future observation.^22^ Let $X_{1},\ldots ,X_{n},X_{n+1}$ be real-valued exchangeable random quantities. We observe $X_{1},\ldots ,X_{n}$ and aim to predict $X_{n+1}$ based on those n observations. The ordered observed values are $x_{\left(1\right)}<x_{\left(2\right)}<\cdots <x_{\left(n\right)}$ and let $x_{\left(0\right)}=-\infty$ and $x_{\left(n+1\right)}=\infty$ , or use known or assumed bounds for the support of the random quantities, say $x_{\left(0\right)}=L$ and $x_{\left(n+1\right)}=R$ .^16^ Then for the future observation $X_{n+1}$ , based on n observations, the assumption $A_{\left(n\right)}$ is^16^:

(1) $$P(X_{n+1}\in (x_{\left(j-1\right)},x_{\left(j\right)}))=\frac{1}{n+1},\;\mathrm{for}\;j=1,2,\ldots ,n+1.$$

This means that $X_{n+1}$ is equally likely to be in any of the intervals created by the ordered observed data. Note that under $A_{\left(n\right)}$ it is assumed that there are no ties. Methods for dealing with ties in general nonparametric statistical methods are presented by Gibbons and Chakraborti.^23^ In the NPI framework, ties can be dealt with by breaking them by a very small amount.^24–26^

The NPI approach can also be used for multiple future observations via the consecutive application of Hill’s assumption $A_{\left(n\right)}$ , $A_{\left(n+1\right)}$ ,…, $A_{\left(n+m-1\right)}$ , which together are denoted by $A_{\left(\cdot \right)}$ .^20^ An ordering $O_{i}$ represents the possible positions of the m>1 future observations relative to the n data observations. There are $\left(\begin{array}{c} n+m\\ n \end{array}\right)$ possible orderings of n data observations and m future observations, and under $A_{\left(\cdot \right)}$ all these orderings are equally likely.^20,27^ Let $S_{j}^{i}$ denote the number of future observations in the interval $I_{j}=(x_{\left(j-1\right)},x_{\left(j\right)})$ given the specific ordering $O_{i}$ , where $i=1,\ldots ,\left(\begin{array}{c} n+m\\ n \end{array}\right)$ and j=1,…,n+1 . Here $s_{j}^{i}$ is a non-negative integer and $\sum_{\;j=1}^{n+1}s_{j}^{i}=m$ . As a consequence of the assumption $A_{\left(\cdot \right)}$ we have the following result, which is central to NPI for multiple future observations:

(2) $$P\left(\overset{n+1}{\underset{\;j=1}{⋂}}\{S_{j}^{i}=s_{j}^{i}\}\right)=P(O_{i})={\left(\begin{array}{c} n+m\\ n \end{array}\right)}^{-1}$$

Any specific ordering only specifies the number of future observations in each interval $I_{j}$ , no assumptions are made about where exactly in $I_{j}$ the future observations will be. In general, uncertainty is often expressed using lower and upper probabilities in the NPI framework. The lower probability for an event E is the proportion of orderings $O_{i}$ for which event E is necessarily true, while the upper probability for E is the proportion of orderings $O_{i}$ for which E can hold.^20^

In this paper, however, we do not compute lower and upper reproducibility probabilities for the t -test for two reasons: First, it is computationally hard to derive such lower and upper probabilities for practical data sets since the number of orderings to consider grows exponentially as the number of the original data points increases. Secondly, computing the minimum and maximum values of the t -test statistic for m future observations with given ordering $O_{i}$ is difficult, because this statistic depends both on the sample mean and variance. Instead, we use NPI bootstrap (NPI-B), which, rather than calculating lower and upper probabilities, tends to provide a value in between which serves well as an indication of the test reproducibility.^28^ NPI-B is based on $A_{\left(\cdot \right)}$ and it follows the concept of all orderings $O_{i}$ being equally likely.^28^ NPI-B differs from Efron’s bootstrap,^29^ mainly as it is developed for prediction, for which it is important that future observations are not restricted to already observed values, while Efron’s bootstrap is aimed at estimating of population characteristics.^18,28^

In the NPI-B method, there are n data observations and interest is in m future observations. Let N denote the number of bootstrap samples. The NPI-B method is as follows:

1. Create n+1 intervals from n ordered observations.
2. Sample an interval with equal probability.
3. From that interval, sample uniformly a value and then add it to the data set.
4. In total sample m further values in the same way to form an NPI-B sample.
5. Create in total N NPI-B samples.

Note that, in this paper, bounded ranges [L,R] for the random quantities are assumed for NPI-B. It is possible to generalise this to sampling from the real-line,^18^ but it does not make a substantial difference to the reproducibility of the considered tests and it can greatly increase computation time. We determine the bounds L and R , based on the sample, as follows: $L=x_{\left(1\right)}-\max_{i}(x_{\left(i\right)}-x_{\left(i-1\right)})$ and $R=x_{\left(n\right)}+\max_{i}(x_{\left(i\right)}-x_{\left(i-1\right)})$ , where i=2,3,…,n .

## NPI reproducibility for pairwise t -test

The NPI reproducibility probability is the probability for the event that, if a test were repeated under identical circumstances and with the same sample size, the same test outcome would be reached. The NPI reproducibility probability does not imply anything about getting the test outcome ‘right’; for that, traditional aspects of hypothesis testing, such as level significance, power and other related post-data metrics, are relevant. This section studies reproducibility for the Student’s t -test for comparison of two groups from the NPI perspective. First, we introduce an algorithm for calculating NPI reproducibility for the t -test for comparison of two groups of data (Algorithm 1). Secondly, the NPI reproducibility is explored though simulations in section ‘Simulations’. Within the simulations, relationships between the reproducibility probability and statistics of the original data (the p -value and Cohen’s d estimate) are studied. As a nonparametric counterpart to the t -test, the WMT can be performed to compare two groups in cases where the normality assumption may not be reasonable. Thus, we briefly investigate reproducibility of the WMT and compare it to reproducibility of the t -test in section ‘NPI reproducibility for WMT’.


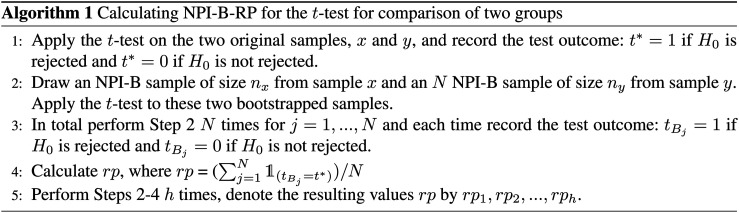


### Algorithm for NPI reproducibility for pairwise t -test

Algorithm 1 uses NPI bootstrap to derive the reproducibility probability for the t -test, indicated by NPI-B-RP. As these values result from the use of the NPI bootstrap methods, they are effectively estimates. The inputs into Algorithm 1 are the two original samples, x and y , their corresponding sample sizes $n_{x}$ and $n_{y}$ , the number of runs h and the number of bootstrapped samples per run N . We apply Algorithm 1 with N=1000 and h=100 .

The algorithm for calculating NPI-B-RP for the t -test has been adopted from the NPI-B-RP for the WMT, which was presented in BinHimd’s thesis,^18^ who briefly investigated NPI reproducibility for the WMT.

### Simulations

In this section, we study the reproducibility probability (NPI-B-RP) for the t -test via simulations, where we calculate the reproducibility using Algorithm 1. The null hypothesis is $H_{0}\;:\;\mu_{x}=\mu_{y}$ and the alternative hypothesis is $H_{1}\;:\;\mu_{x}>\mu_{y}$ , the level of significance is α=0.05 . We simulate data both under $H_{0}$ and under $H_{1}$ . Under $H_{0}$ we generate data from the normal distribution with mean 0 and standard deviation 1 for both groups. Under $H_{1}$ we generate data from two normal distributions with different means, $\mu_{x}=1$ and $\mu_{y}=0$ , but both with standard deviation 1 . Further simulations were performed for different values of the means and standard deviations under $H_{1}$ , these all led to similar results as for the case presented here.

The inputs for the simulation study are as follows: the sample size n=6,10,20 ; means $\mu_{1}$ , $\mu_{2}$ and standard deviations $\sigma_{x}$ and $\sigma_{y}$ are as given in the previous paragraph; and the number of runs per simulation N=200 . For each run, one sample of size n is generated from each of these normal distributions, the t -test is performed on these two samples and the p -value is computed, and NPI-B-RP for the t -test is calculated using Algorithm 1. We also consider Cohen’s d for the tests; this is an often used measure of the standardised effect size for comparisons of two samples. Cohen’s d is given by the following equation^30^:

$$d=\frac{\left(\bar{x}-\bar{y}\right)}{s}$$

where s is the pooled sample standard deviation. As two simulated samples in pairwise tests in this paper are always of the same size, and the samples in the pharmaceutical scenario in section ‘NPI reproducibility for t -test applied to a pharmaceutical test scenario’ are nearly of the same size while their standard deviations are similar, we just use as pooled sample standard deviation the average of the two individual sample standard deviations $s_{x}$ and $s_{y}$ , that is

$$s=\sqrt{\frac{s_{x}^{2}+s_{y}^{2}}{2}}$$.

**Figure 1.**
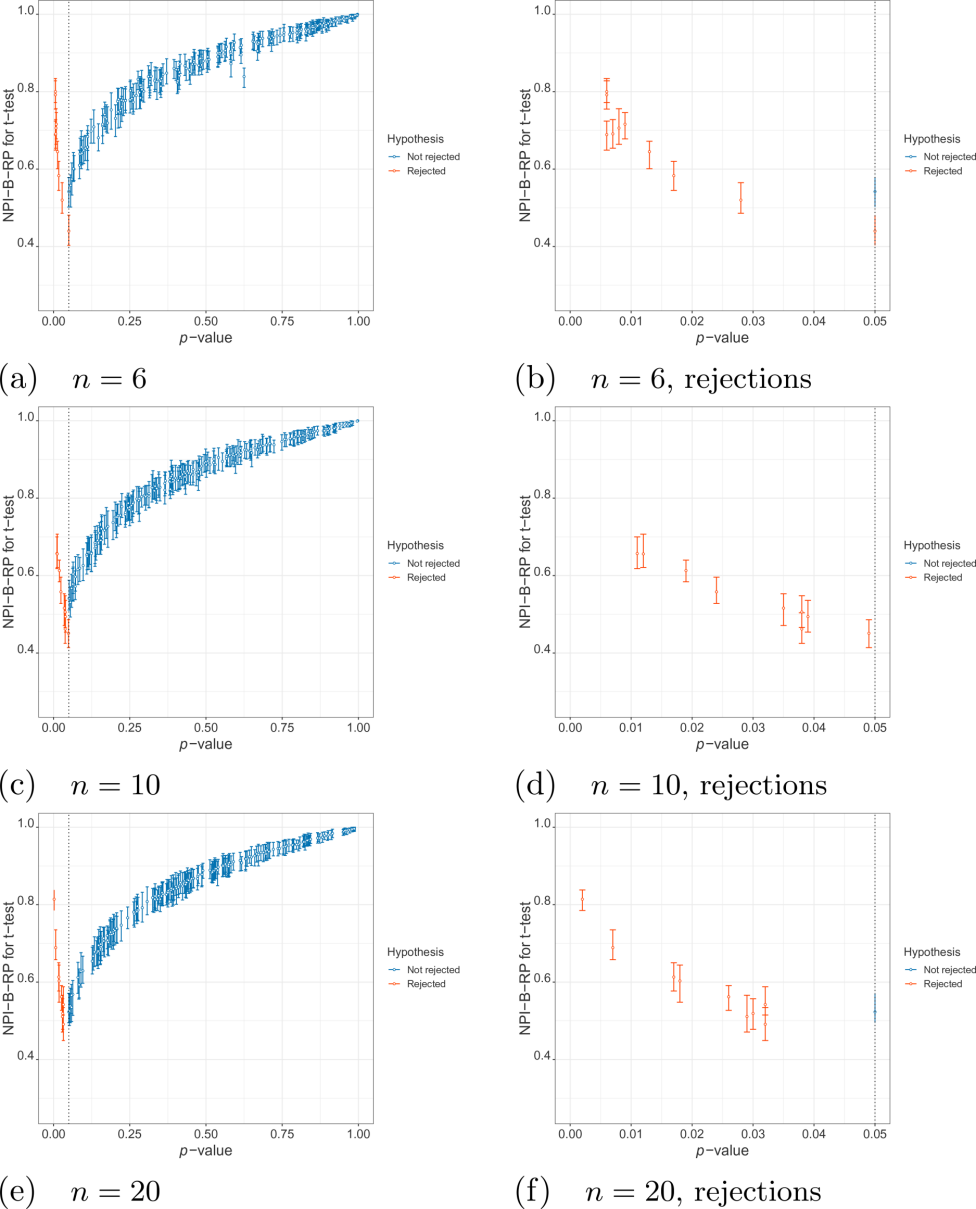
Simulations under $H_{0}$ : values of nonparametric predictive inference bootstrap reproducibility probability (NPI-B-RP) (minimal, mean and maximal) for the t -test vs p -value.

**Figure 2.**
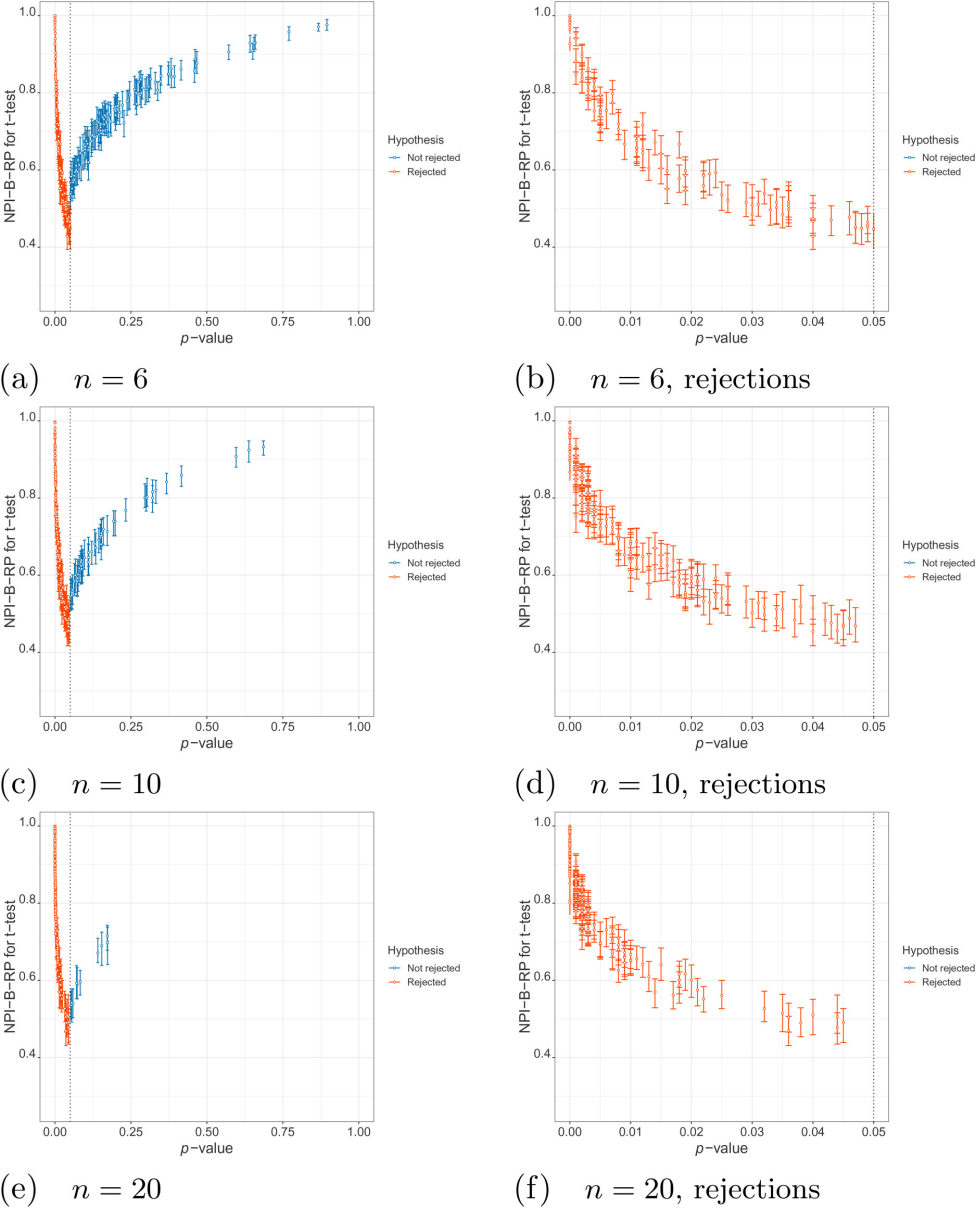
Simulations under $H_{1}$ : values of nonparametric predictive inference bootstrap reproducibility probability (NPI-B-RP) (minimal, mean and maximal) for the t -test vs p -value.

First, we examine the relationship between NPI-B-RP and the p -value for the t -test in the simulations. Figure 1 (simulations under $H_{0}$ ) and Figure 2 (simulations under $H_{1}$ ) display plots of these metrics for the three different sample sizes, with separate plots for the rejection cases only ( p -value less than 0.05 ). It is clear that, as expected, reproducibility is the lowest close to the test threshold, so if the p -value is close to α=0.05 , In such cases, NPI-B-RP tends to be lower in case of rejection (red cases in the figures) than for non-rejection (blue cases). Low values of NPI-B-RP are worrying from a practical perspective, in particular in case $H_{0}$ is rejected with the p -value only just below the level of significance, because many experiments are explicitly designed with the aim to find evidence supporting $H_{1}$ . NPI-B-RP tends to increase when the p -value moves away from α=0.05 , which is also as expected. Similar patterns have been seen in applications of NPI reproducibility for several other test scenarios.^6,21^ For the simulations under $H_{1}$ , increasing n leads to fewer cases with larger p -values, which simply results from the test becoming more powerful for larger n . As a consequence, reproducibility for most non-rejection cases for larger n becomes relatively lower compared to non-rejection cases for small n , when data are sampled under $H_{1}$ .

**Figure 3.**
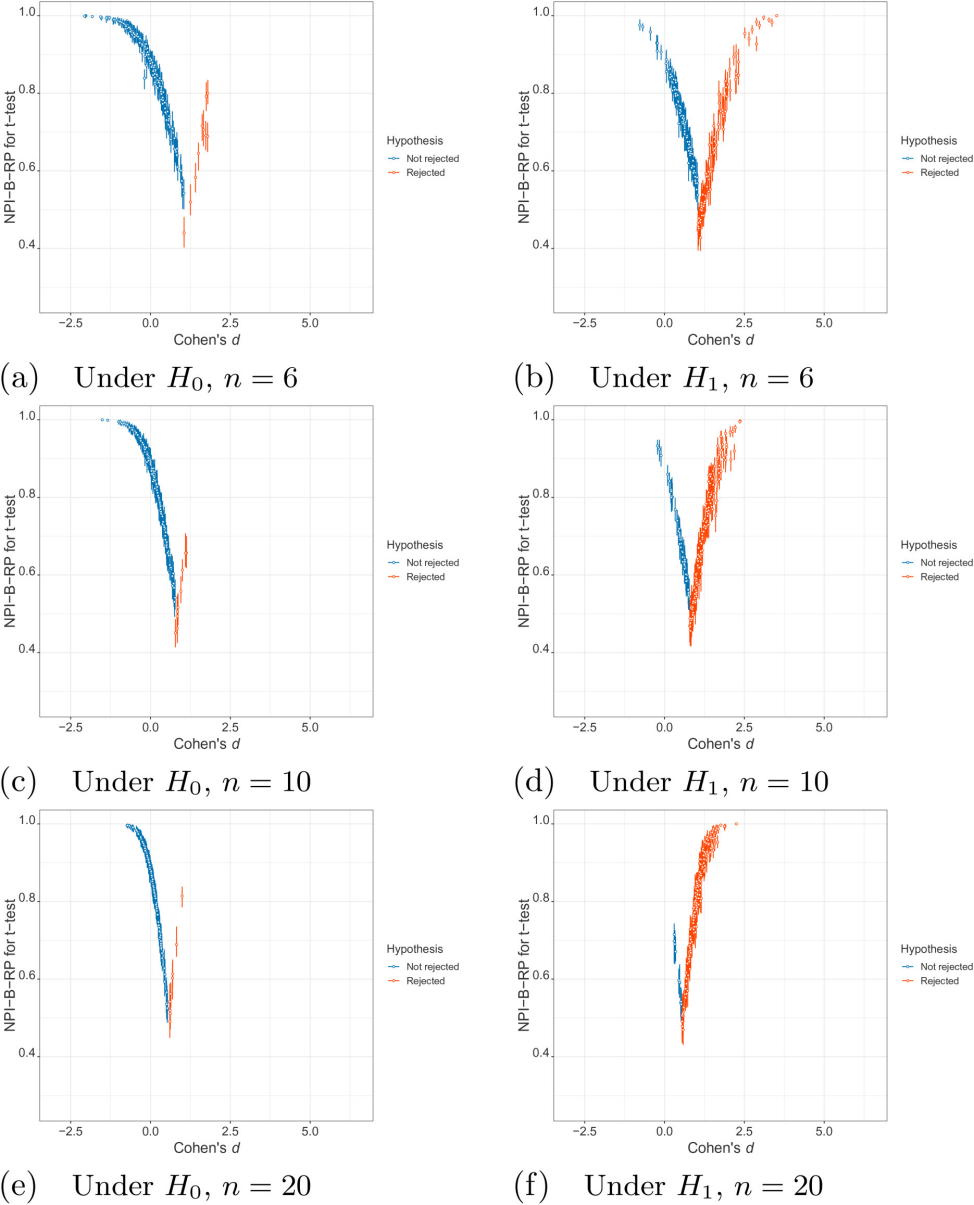
Simulations under $H_{0}$ and $H_{1}$ : values of nonparametric predictive inference bootstrap reproducibility probability (NPI-B-RP) (minimal, mean and maximal) for the t -test vs Cohen’s d .

Secondly, we explore the relationship between NPI-B-RP and Cohen’s d . Figure 3 shows the plots of these two metrics for simulations under $H_{0}$ and $H_{1}$ . In Figure 3, there is a V-shaped pattern: both for the rejection cases (right side of the V-shape, in red) and the non-rejection cases (left side of the V-shape, in blue), the NPI reproducibility of the t -test tends to increase when Cohen’s d moves away from the area where the V-shape has the lowest point. The patterns are similar across the different distribution parameters and sample sizes, where the range of the values of Cohen’s d becomes a bit smaller for larger sample sizes due to the reduced variability of the sample means.

Finally, we study variability of NPI-B-RP by applying Algorithm 1 several times for the same two datasets. The resulting outputs varied very little among the different applications, with the means of the values $rp_{1},\ldots ,rp_{100}$ differing only in the third decimal. As this mean of the rp -values can be considered to best present the NPI reproducibility of the test, this rather minimal variability suggests that the choices N=1000 and h=100 are appropriate to ensure that our inferences are accurate.

### NPI reproducibility for Wilcoxon-Mann-Whitney test 

**Figure 4.**
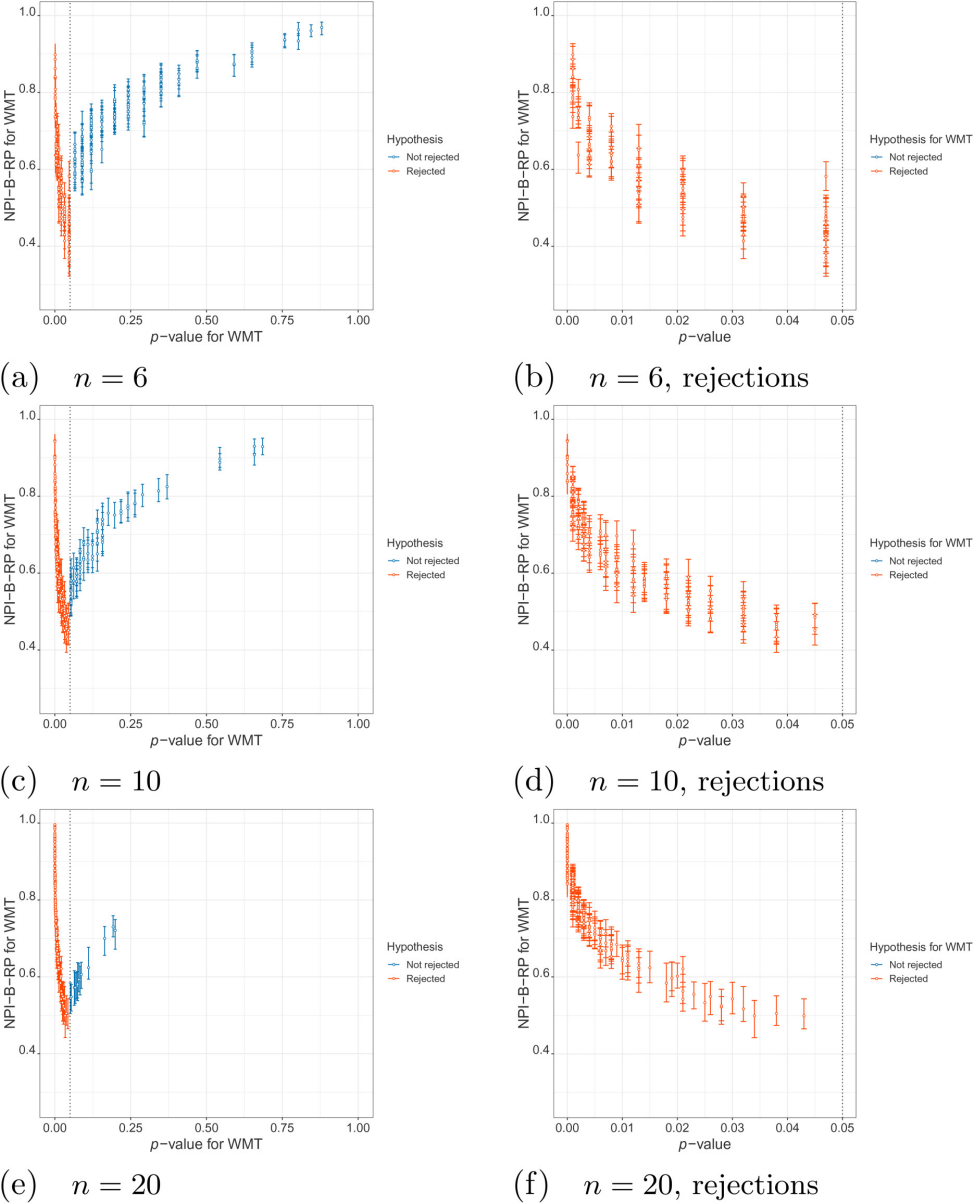
Simulations under $H_{1}$ : values of NPI-B-RP (minimal, mean and maximal) for the WMT versus p -value.

It is of interest to compare NPI-B-RP for the t -test with NPI-B-RP for the Wilcoxon-Mann-Whitney test (WMT),^31^ an often used nonparametric counterpart to the t -test. This is straightforward by replacing the t -test by the WMT in Algorithm 1. Figure 4 shows plots for the NPI-B-RP for the WMT and the p -values for the WMT for simulations under $H_{1}$ . These show a similar relationship between the reproducibility probability and the p -value as for the t -test in Figure 2, with however fewer different p -values being possible due to the WMT being based on the ranks. Comparison of the reproducibility of these two tests with simulated data under $H_{0}$ also led to very similar results, these are not reported here.

## NPI reproducibility for t -test applied to a pharmaceutical test scenario

This section presents the application of NPI-B-RP for the pairwise t -tests, as presented in section ‘NPI reproducibility for pairwise t -test’, to a pre-existing dataset from an internal preclinical study assessing the optimal dose of a drug. No new experiments were carried out and the original statistical analysis framework for the experiment was adopted. Section ‘Pharmaceutical test scenario’ introduces the motivating pharmaceutical test scenario. NPI reproducibility for the pairwise comparisons in this scenario is presented in section ‘NPI reproducibility for the pairwise tests for the pharmaceutical test scenario’.

### Pharmaceutical test scenario

**Figure 5.**
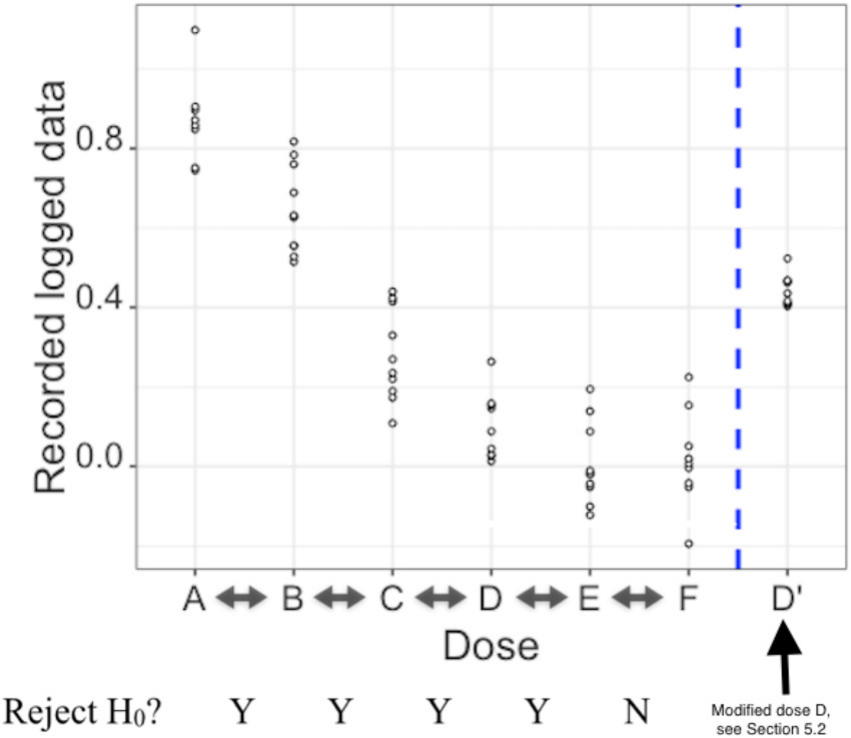
Log transformed data for each dose and outcomes of the pairwise comparisons (D’ only used in section ‘Further illustration of reproducibility of the final decision’).

The experiment assesses six concentrations of a drug; A is the control group and B-F are groups given increasing concentrations of the drug. For each group, there is one measurement available for each individual. The measurement is such that the lower the recorded value is, the better the drug performs at that concentration. The data has been log transformed to meet the t -test assumption of normality; they are presented in Table 1 and Figure 5.

**Table 1.** Log transformed data for each dose (D’ replaces D in section ‘Further illustration of reproducibility of the final decision’).

Dose						
A	B	C	D	E	F	D’
0.7450	0.5148	0.1088	0.0133	−0.1221	−0.1946	0.4033
0.7513	0.5280	0.1732	0.0265	−0.1010	−0.0520	0.4087
0.8484	0.5546	0.1896	0.0302	−0.0519	−0.0417	0.4103
0.8584	0.5553	0.2202	0.0444	−0.0436	−0.0039	0.4163
0.8728	0.6265	0.2352	0.0882	−0.0200	0.0076	0.4354
0.8964	0.6315	0.2697	0.1461	−0.0182	0.0196	0.4624
0.9053	0.6890	0.3298	0.1545	−0.0104	0.0512	0.4665
1.0981	0.7605	0.4150	0.1585	0.0879	0.1540	0.4684
	0.7843	0.4234	0.2638	0.1390	0.2247	0.5232
	0.8173	0.4401		0.1945		

Five pairwise comparisons are carried out between adjacent concentrations of the drug (A vs. B, B vs. C, C vs. D, D vs. E, E vs. F). For each pairwise comparison, the question of interest is if the dose with higher concentration is performing better than the dose with lower concentration. In each pairwise comparison, the upper-sided equal variance t -test is applied. Let $\mu_{H}$ denote the population mean for the dose with higher concentration and $\mu_{L}$ the population mean for the dose with lower concentration. The null hypothesis is $H_{0}\;:\;\mu_{L}=\mu_{H}$ and the alternative hypothesis is $H_{1}\;:\;\mu_{L}>\mu_{H}$ . The significance level α is equal to 0.05. For each pairwise comparison, the test outcome is either to reject (Y) or to not reject (N) the null hypothesis.

The results of multiple pairwise comparisons for the data presented in Table 1 are YYYYN, indicating that the null hypotheses are rejected for all pairwise comparisons except for last one, E vs. F. As seen in Figure 5, as the dose increases, the measurements tend to decrease until dose E.

Note that the WMT leads to the same test outcomes for all these pairwise comparisons.

### NPI reproducibility for the pairwise tests for the pharmaceutical test scenario

In this section, Algorithm 1 (from section ‘NPI reproducibility for pairwise t -test’) is applied to the test scenario described in section ‘Pharmaceutical test scenario’ and conclusions regarding reproducibility are drawn. The Algorithm 1 outputs and the statistics of the original test for all pairwise comparisons are presented in Table 2. We consider the mean value of the outputs as the best indication of NPI reproducibility, we also refer to this mean as the NPI-B-RP value.

**Table 2.** Statistical and reproducibility analysis for the pairwise comparisons.

	Statistics of the original data				Algorithm 1 output					
Pair	Reject?	p -value	Effect Size	Cohen’s d	t -test			WMT		
					min	mean	max	min	mean	max
A vs. B	Yes	0.0003	0.226	2.041	0.917	0.937	0.954	0.882	0.902	0.927
B vs. C	Yes	0.0000	0.366	3.213	0.999	1.000	1.000	0.999	1.000	1.000
C vs. D	Yes	0.0007	0.178	1.753	0.841	0.880	0.904	0.821	0.862	0.890
D vs. E	Yes	0.0191	0.097	1.038	0.552	0.586	0.622	0.566	0.606	0.642
E vs. F	No	0.5977	−0.013	−0.115	0.885	0.911	0.928	0.917	0.935	0.958

First, we consider what conclusions about NPI-B-RP can be directly made from the pharmaceutical test scenario. The pairwise comparison E vs. F has high NPI-B-RP value, 0.911. This means that if the test were repeated under identical circumstances and with the same sample sizes, then the same test outcome would be reached with estimated probability 0.911. By comparison, the NPI-B-RP value for the pairwise comparison D vs. E is 0.586. It is up to the decision makers to consider the NPI-B-RP values alongside other statistical information and inferences, such as the effect size and power, in order to decide on the trustworthiness of the test results.

**Figure 6.**
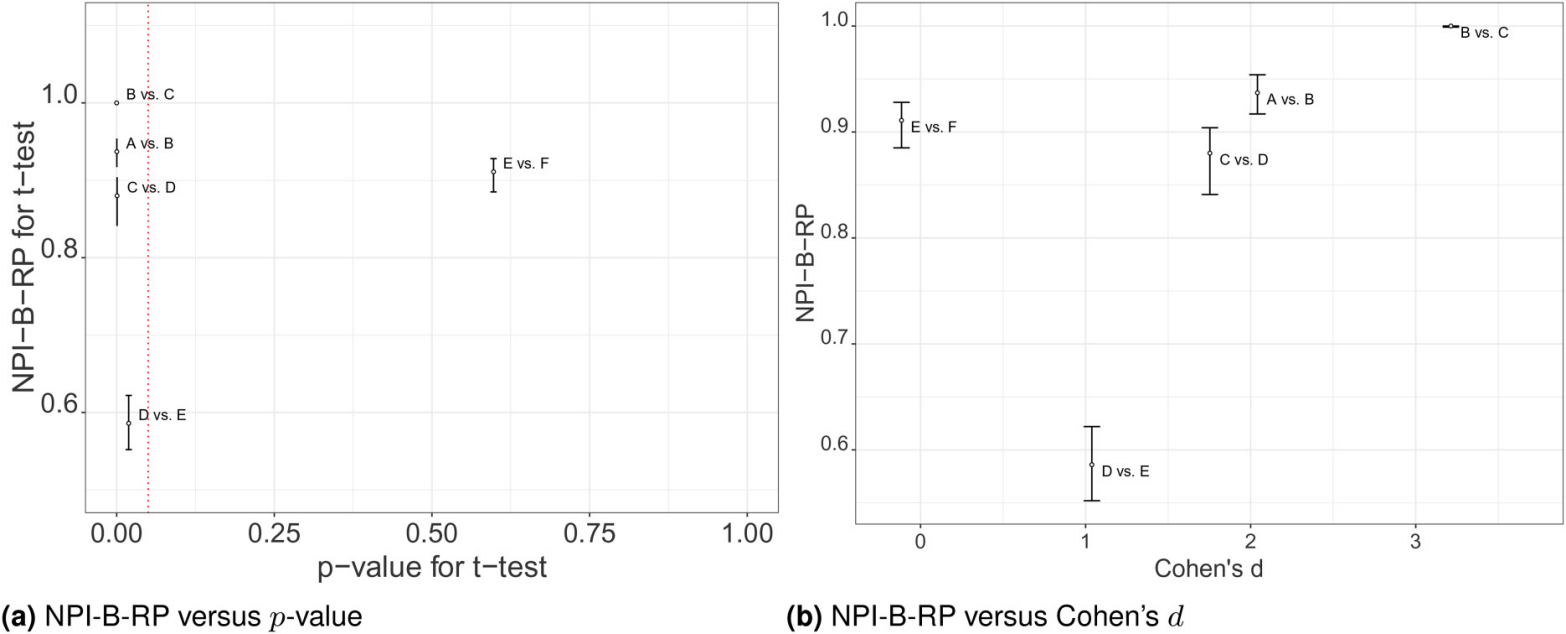
Comparing values of nonparametric predictive inference bootstrap reproducibility probability (NPI-B-RP) (minimal, mean and maximal) for t -test to the statistics of the original test. (a) NPI-B-RP versus p -value. (b) NPI-B-RP versus Cohen’s d .

Secondly, we explore how the NPI reproducibility values relate to statistics of the t -test applied to the original data, these statistics are also displayed in Table 2. Note that these tests are pairwise comparisons where we do not yet take into account that multiple tests are performed simultaneously. Effect Size is the difference between the respective sample means; as Cohen’s d is closely related to it, and the relationships between NPI-B-RP for the t -test and either the Effect Size or Cohen’s d are very similar; thus, we only consider Cohen’s d in the following discussion. Figure 6 illustrates the relationship between NPI-B-RP for the t -test, indicating the minimum, mean and maximum values of the NPI-B-RP output of Algorithm 1, for each of the pairwise comparisons, and the p -values and Cohen’s d . There are some clear patterns: For example, NPI-B-RP is smallest for the pairwise comparison D vs. E, where the p -value is closest to the threshold value 0.05 and Cohen’s d is small. A further observation is that high NPI-B-RP values are obtained for several of the pairwise comparisons, both for some cases where the null hypothesis is rejected, in particular for the comparison B vs. C, and for the comparison E vs. F where the null hypothesis is not rejected. For B vs. C, the p -value is very small compared to α=0.05 and Cohen’s d is very large, as Cohen’s d greater than 0.8 is typically considered to be large.^30^ For E vs. F, the p -value is very large compared to α=0.05 and Cohen’s d is negative. We conclude that our observations about NPI-B-RP for the pharmaceutical test scenario are consistent with the observations made in section ‘Simulations’. The key observations are: NPI-B-RP is low when the p -value is close to the level of significance α . For non-rejection cases, even when the p -value is much greater than α , NPI-B-RP stays a bit below 1.

**Figure 7.**
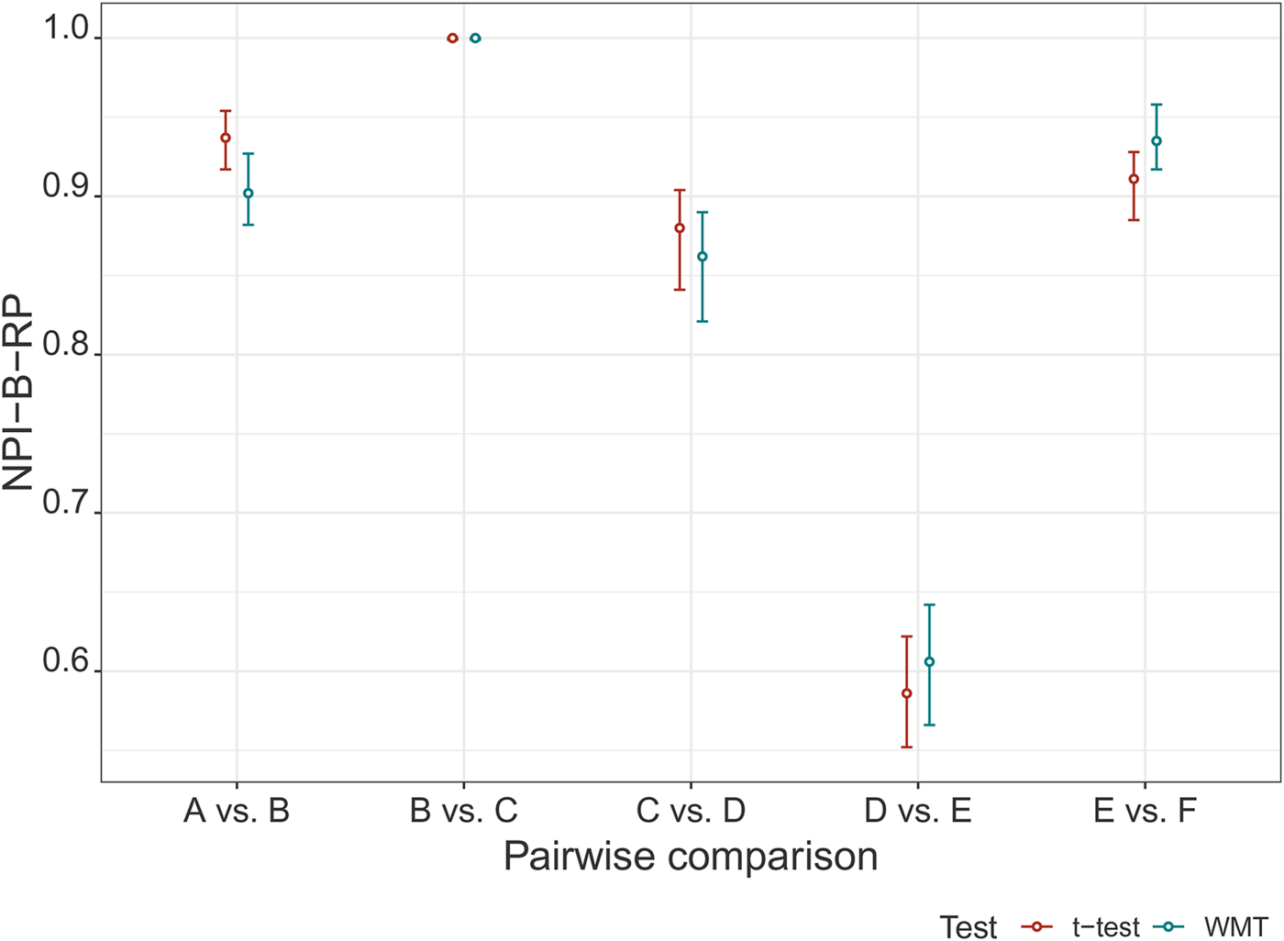
Values of NPI-B-RP (minimal, mean and maximal) for t -test and WMT.

Finally, we compare NPI-B-RP for the t -test and for the WMT (Figure 7). The NPI-B-RP values for both tests for this case study are quite similar. This may be due to the fact that the log-transformed data used can reasonably be assumed to be normally distributed. This conclusion also agrees with the conclusions from the simulation study (section ‘NPI reproducibility for WMT’).

## Reproducibility of the final decision based on multiple pairwise comparisons

In section ‘NPI reproducibility for pairwise t -test’, we introduced NPI-B-RP for the t -test for the comparison of two groups and in section ‘NPI reproducibility for t -test applied to a pharmaceutical test scenario’ we presented NPI-B-RP for pairwise comparisons in a pharmaceutical test scenario. However, in this test scenario, the final choice of a particular dose is based on the multiple pairwise comparisons. This section explores the NPI-B-RP of this final decision and presents a general algorithm for calculating such reproducibility.

In a case involving multiple pairwise comparison tests, it is important to consider how the final decision is made, and which dose is finally selected. We consider the scenario that the decision maker selects the smallest dose for which, in the pairwise comparisons above, the null hypothesis of no difference between the results for this dose and the next larger dose, is not rejected. In the presented test scenario, this leads to dose E being chosen, and only the actual outcomes of the five pairwise tests, which we can present as YYYYN, leads to this final decision.

In section ‘Algorithm for NPI reproducibility for the final decision’, we present the general algorithm for calculating reproducibility of the final decision, and we apply this algorithm to the test scenario from section ‘Pharmaceutical test scenario’. In section ‘Further illustration of reproducibility of the final decision’, we modify the data from the test scenario in order to illustrate and explore reproducibility of the final decision.


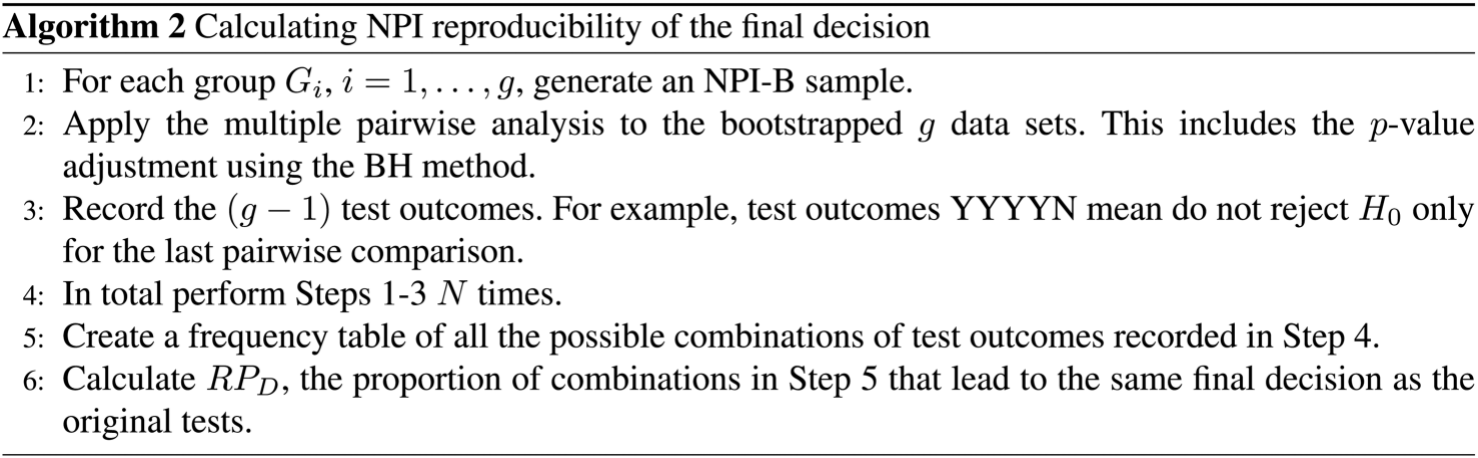


### Algorithm for NPI reproducibility for the final decision

Algorithm 2 presents a step-by-step general method for calculating NPI-B-RP of the final decision. The number of groups in the multiple pairwise comparison is denoted by g . Similarly to Algorithm 1, Algorithm 2 uses NPI bootstrap with finite intervals, as introduced in section ‘Nonparametric predictive inference and bootstrap’. So for each group, $G_{i}$ , i=1,…,g , finite end points for the range of the possible values need to be selected. The sample sizes of the bootstrap samples are the same as of the original data. The reproducibility for the final decision, denoted by $RP_{D}$ , is defined as the proportion of all the combined g−1 test outcomes leading to the same final decision as the original tests. In order to account for the fact that the five tests are run simultaneously, the p -values are adjusted for multiple testing using the Benjamini and Hochberg (BH) procedure^32^ to control the false discovery rate. The adjusted *p*-values for each pairwise comparison are A vs. B: 0.0007 ; B vs. C: $2.7\times 10^{6}$ ; C vs. D: 0.0012; D vs. E: 0.0239; E vs. F: 0.5977. This procedure strives to decrease the proportion of false positives. In the test scenario, after the p -value adjustment, the test decision outcomes are still YYYYN.

We apply Algorithm 2 to the pharmaceutical test scenario from section ‘Pharmaceutical test scenario’ with g=6 groups. We set N=1000 and the final decision is based on the test results YYYYN, and so dose E is chosen because there is no significant indication that dose F is better than dose E. Algorithm 2 leads to two different types of outcome: A frequency table (Step 5) which provides all the combinations of test outcomes reached in N runs of Step 1-3, and the value of $RP_{D}$ (Step 6), which is the proportion of all combinations of test outcomes that lead to the original test decision.

**Figure 8.**
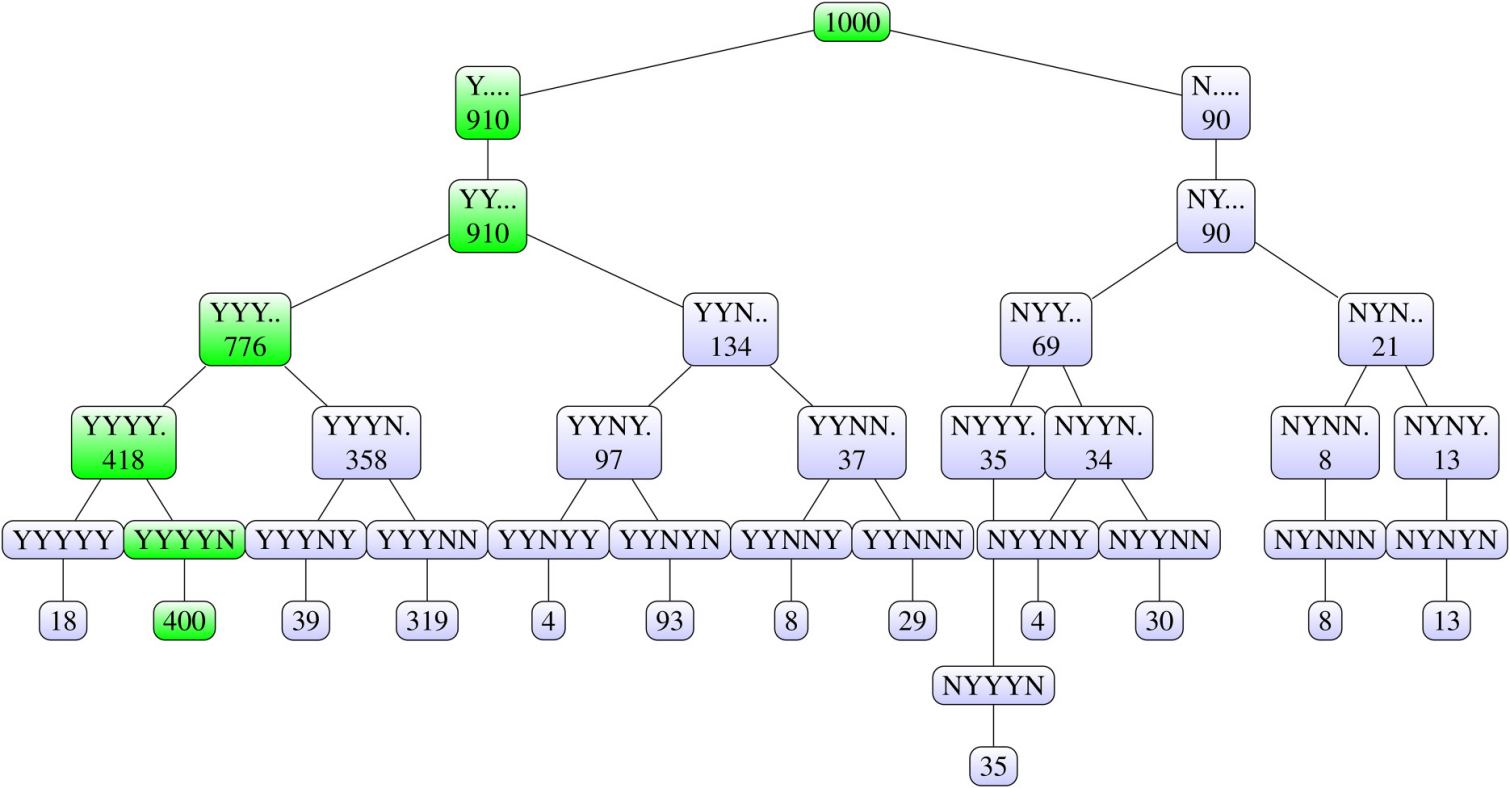
Tree diagram for reproducibility of the final decision for original test scenario (outputs of Step 5 of Algorithm 2).

For this particular dataset and final decision rule, the $RP_{D}$ for an identical final decision (Step 6 of Algorithm 2) is 0.400, which is a relatively low value compared to the NPI-B-RP values for the pairwise comparisons as derived in section ‘NPI reproducibility for the pairwise tests for the pharmaceutical test scenario’. A more nuanced way of exploring the Algorithm 2 outputs is obtained by considering a reproducibility tree, which shows all possible combinations of the g−1 test outcomes occurring in the frequency table. For the data set given in Table 1, there are 32 possible combinations of the 5 test outcomes. Not all combinations of test outcomes are generated by Algorithm 2 on this dataset. Table 3 presents all the combinations of test outcomes and their frequencies. Figure 8 shows the reproducibility tree for the test scenario. The top node represents the 1000 runs of Steps 1-3 in Algorithm 2. This node splits into two nodes: Y ⋯ . all possible test outcomes where in the first pairwise comparison the null hypothesis was rejected, each dot represents a following pairwise comparison with any possible test outcome; and N ⋯ . all combinations of tests outcomes where in the first pairwise comparison the null hypothesis was not rejected. These branches again split, each into two, depending on the conclusion of the second pairwise comparison. For example, YY ⋯ means that the first and second pairwise comparisons led to rejection of the respective null hypothesis. The same pattern is followed up to the last pairwise comparison.

**Table 3.** Frequency table (Step 5 of Algorithm 2).

Combination of test outcomes	Occurrence
YYYYY	18
YYYYN	400
YYYNY	39
YYYNN	319
YYNYY	4
YYNYN	93
YYNNY	8
YYNNN	29
NYYYN	35
NYYNY	4
NYYNN	30
NYNNN	8
NYNYN	13

The most frequent output is YYYYN, which is the same as the original test results and leads to dose E being chosen. The branch leading to this final decision is highlighted. The second most frequent output is YYYNN, leading to dose D being chosen. The fact that YYYNN is the second most frequent output can be explained by the relatively small NPI-B-RP value for the pairwise comparison between doses D and E.

We repeated Algorithm 2, with N=1000 , ten times for this scenario. The resulting reproducibility trees were the same, only the numbers differed slightly, the $RP_{D}$ values, so the proportion of runs leading to the same output YYYYN, were: 0.370, 0.376, 0.388, 0.400, 0.402, 0.403, 0.410, 0.412, 0.415, 0.424. By comparison, the NPI-B-RP values calculated on different separate runs of Algorithm 1 differ in the third decimal. Although small, the variability in these reproducibility probabilities is larger than for the individual pairwise comparisons, this is due to the use of multiple pairwise comparisons to determine the reproducibility of the final decision.

### Further illustration of reproducibility of the final decision

**Figure 9.**
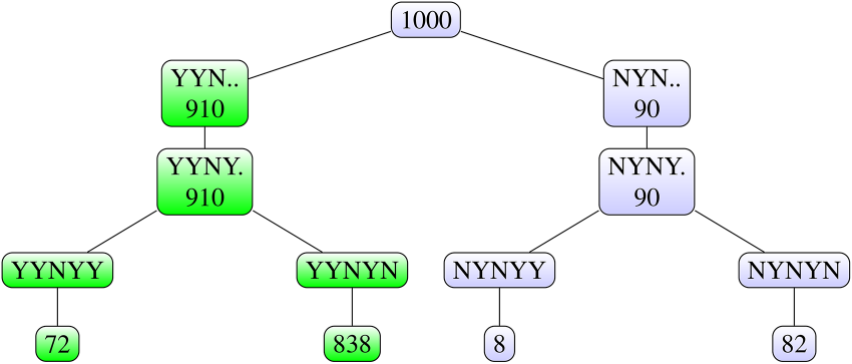
Illustration of the final decision rule: Tree diagram for reproducibility of the final decision for the modified data (outputs of Step 5 of Algorithm 2).

If we follow the final decision rule for the test scenario data, only one combination of the pairwise test results, namely YYYYN, leads to the choice of dose E. To better illustrate the concept of reproducibility of the final decision, we change the data for dose D by adding 1.5 to all the data points before they are log transformed, the resulting values are denoted by D’ in Table 1 and Figure 5. This leads to the pairwise test outcomes YYNYN, and the final decision would be to choose dose C, since dose D does not do better than dose C. To determine the reproducibility of the final decision, we again apply Algorithm 2 to the test scenario with these modified data (Figure 9). Now there are four combinations of test outcomes that lead to the same final decision to choose dose C: YYNYN (the original test outcome), YYNYY, YYNNY and YYNNN. The reproducibility of the final decision is derived as the proportion of all simulation runs in which one of these four combinations of test outcomes occurs. As the combinations YYNNY and YYNNN did not occur, the reproducibility of the final decision for the modified data is derived by summing the proportions of runs with outcomes YYNYY and YYNYN, leading to 0.910, as highlighted in Figure 9. This simulation was also repeated ten times, and the results were very similar, with $RP_{D}$ values 0.894, 0.910, 0.911, 0.917, 0.917, 0.917, 0.919, 0.919, 0.919, 0.922. In all these simulations, the resulting reproducibility trees were the same, with only small differences in the numbers.

## Concluding remarks

NPI reproducibility provides an inference method for the probability for the event that, if a test would be repeated under identical circumstances and with the same sample size, the same test outcome would be reached. This paper contributes to the development of NPI reproducibility by exploring the reproducibility for the two-sample Student’s t -test, which is widely used in practice. First, the reproducibility for the t -test has been studied via simulations, followed by application to such tests in a pharmaceutical scenario. Secondly, reproducibility for a final decision based on multiple pairwise t -tests has been investigated.

We explored the reproducibility of the pairwise t -test and investigated the relationships between NPI reproducibility and two common test statistics, the p -value and the Cohen’s d . As the p -value approaches the significance level α , the NPI reproducibility decreases, and for p -values close to α the NPI reproducibility is typically lower in case of rejection of the null-hypothesis than for non-rejection. This relation also held when we compared the reproducibility and Cohen’s d , and further simulations, beyond the cases presented in this paper and with other input parameters, led to similar results. We also compared reproducibility of the t -test and the WMT in our simulations and for the pharmaceutical test scenario, the results were similar. This might be due to the fact that the considered data could, after transformation, reasonably be assumed to come from a normally distributed population, and the data in the simulation study were generated from normal distributions. More detailed investigation of differences in reproducibilities of these two tests, for example for data from skewed distributions, is a topic for future research.

The NPI reproducibility for the pairwise t -tests can provide useful insights for practical applications. For example, in the pharmaceutical test scenario one of the pairwise comparisons had low reproducibility, so it might be advisable to explore the comparison of those two groups in more detail, possibly by additional experiments. NPI reproducibility can be used in conjunction with other test statistics, such as the p -value and the Cohen’s d , to support the decision process based on the data and tests. Such use of NPI reproducibility in practical decision making is left as an important topic for future research.

In the pharmaceutical scenario considered in this paper, multiple comparisons are performed and their test results lead to a final decision on an appropriate dose. It is therefore also important to consider the reproducibility of this final decision; and one could say that this is the most important outcome of the combined hypothesis tests. We introduced an algorithm for deriving the NPI reproducibility of this final decision, this has not previously been considered in the literature. For the presented pharmaceutical test scenario, the reproducibility of the final decision is smaller than the reproducibilities for all the pairwise comparisons on which the final decision is based. This is a logical consequence of using multiple pairwise comparisons to reach the final decision. Low reproducibility of the final decision should be taken into account by decision makers, investigating possible further actions to improve this situation is also left for future research.

Related to this paper, there are many more topics for future research. The study of the sensitivity of the reproducibility calculations to the choice of the left and the right bound of the support of the finite bootstrap could be investigated. The reproducibility in this paper is expressed with the use of precise probabilities, whereas classical NPI uses the more general concept of imprecise probability to quantify uncertainty, hence leading to lower and upper reproducibility probabilities. Deriving NPI lower and upper probabilities for the t -test is an interesting topic for further research. Coolen and Marques^33^ carried out research on determining estimates for NPI-RP through sampling of orderings for likelihood test; this method could be explored for the tests in this paper if the NPI lower and upper reproducibility probabilities can be computed or approximated. The main challenge is to apply NPI reproducibility to many real-world test scenarios and to use it as input into actual decision processes. Follow-up actions in case of low reproducibility are also important and research into this has not yet been reported in the literature.
